# Epidemiology and Outcomes in Critically Ill Patients with Human Immunodeficiency Virus Infection in the Era of Combination Antiretroviral Therapy

**DOI:** 10.1155/2017/7868954

**Published:** 2017-02-27

**Authors:** Shannon L. Turvey, Sean M. Bagshaw, Dean T. Eurich, Wendy I. Sligl

**Affiliations:** ^1^Division of Infectious Diseases, Faculty of Medicine and Dentistry, University of Alberta, Edmonton, AB, Canada; ^2^Department of Critical Care Medicine, Faculty of Medicine and Dentistry, University of Alberta, Edmonton, AB, Canada; ^3^School of Public Health, University of Alberta, Edmonton, AB, Canada

## Abstract

*Purpose.* The impact of critical illness on survival of HIV-infected patients in the era of antiretroviral therapy remains uncertain. We describe the epidemiology of critical illness in this population and identify predictors of mortality.* Materials and Methods.* Retrospective cohort of HIV-infected patients was admitted to intensive care from 2002 to 2014. Patient sociodemographics, comorbidities, case-mix, illness severity, and 30-day mortality were captured. Multivariable Cox regression analyses were performed to identify predictors of mortality.* Results.* Of 282 patients, mean age was 44 years (SD 10) and 169 (59%) were male. Median (IQR) CD4 count and plasma viral load (PVL) were 125 cells/mm^3^ (30–300) and 28,000 copies/mL (110–270,000). Fifty-five (20%) patients died within 30 days. Factors independently associated with mortality included APACHE II score (adjusted hazard ratio [aHR] 1.12; 95% CI 1.08–1.16; *p* < 0.001), cirrhosis (aHR 2.30; 95% CI 1.12–4.73; *p* = 0.024), coronary artery disease (aHR 6.98; 95% CI 2.20–22.13; *p* = 0.001), and duration of HIV infection (aHR 1.07 per year; 95% CI 1.02–1.13; *p* = 0.01). CD4 count and PVL were not associated with mortality.* Conclusions.* Mortality from an episode of critical illness in HIV-infected patients remains high but appears to be driven by acute illness severity and HIV-unrelated comorbid disease rather than degree of immune suppression.

## 1. Introduction

Human immunodeficiency virus (HIV) infection was historically perceived as an independent marker of poor prognosis among patients with critical illness [1–4]. However, the introduction of combination antiretroviral therapy (cART) in 1996 changed the landscape of HIV care dramatically, with substantial reductions in morbidity and mortality in HIV-infected patients [5–9]. HIV infection is now a chronic disease rather than a terminal illness [7, 8, 10]. In the era of cART, the incidence of opportunistic infections (OIs) and HIV-associated malignancies has declined, rates of hospital admission have fallen, and life expectancy has increased [7, 11–13].

However, despite improvements in HIV care, ICU admission rates in HIV-infected patients have stayed stable or increased [7, 12]. HIV-unrelated comorbidities now account for the majority of ICU admissions in this population [11]. In particular, sepsis and complications of chronic liver disease have increased in relative importance [5]. Chronic inflammation and subtle immune dysregulation despite cART may contribute to an increased risk of sepsis in this population [14]. Short-term mortality rates in HIV-infected patients admitted to ICU have fallen in the cART era [9, 15], but their outcomes relative to the general population are conflicting. While a recent study found that HIV status did not affect outcomes in critically ill patients [16], other data suggest mortality in critically ill HIV-infected patients continues to exceed that of HIV-uninfected ICU patients [11, 12]. To date, published data on the epidemiology of critical illness in Canadian HIV-infected patients, as well as their outcomes, are limited.

The aim of our study was to describe the epidemiology of critical illness among HIV-infected patients in Edmonton, Alberta, and to identify factors associated with mortality in this population. We hypothesized that higher illness severity and lower CD4 cell count would be associated with higher 30-day mortality.

## 2. Materials and Methods

The Research Ethics Board at the University of Alberta approved the study and obviated the need for informed consent (study number Pro00026353). STROBE guidelines for reporting of observational studies were followed [17].

### 2.1. Study Design, Setting, and Population

This retrospective population-based cohort study was conducted in Edmonton, Alberta, an urban Canadian center with a population of approximately 1.3 million and a large northern catchment area. The city has five closed general medical/surgical ICUs: two academic/tertiary care ICUs and three community ICUs. The region admits approximately 3500 ICU patients per year.

All adult (aged 17 years or greater) HIV-infected patients admitted to ICU from July 1, 2002, to July 31, 2014, were included. We did not distinguish between patients admitted to the ICU directly from the community and those transferred from a hospital ward. Patients were identified by electronically cross-referencing ICU admission data with our regional HIV database. For each ICU admission, the medical record was reviewed and patient data extracted.

### 2.2. Data Collection and Definitions

Sociodemographic data included age, sex, ethnicity, and HIV risk factors. Comorbidity data included alcohol/drug addiction, cirrhosis, coronary artery disease (CAD), chronic obstructive pulmonary disease (COPD), psychiatric disease, previous OI, history of malignancy, and viral coinfection. Hepatitis C virus (HCV) coinfection was defined as HCV RNA positivity or anti-HCV IgG positivity without an available HCV RNA. Hepatitis B virus (HBV) infection was defined as surface antigen (HBsAg) positivity.

HIV disease severity factors included the most recent CD4 count (cells/mm^3^, percentage [%]) and HIV plasma viral load (PVL, in copies/mL) prior to admission to ICU. To account for changes in assay sensitivity over the study period a cut-off of <200 copies/mL was used to define suppressed PVL. Where CD4 count and PVL had not been performed within the preceding six months, the first CD4 count and PVL recorded after ICU admission were used.

Patients who held active prescriptions for cART at the time of ICU admission were categorized as taking cART, unless known to be nonadherent. cART was defined as an antiretroviral (ARV) regimen consisting of at least three drugs from at least two classes. Seroconversion date and duration of HIV infection were recorded. A new diagnosis of HIV was defined as newly positive testing during the index hospital admission prior to discharge from ICU.

Admission diagnosis (characterized as respiratory failure, sepsis, trauma, overdose, or other) was recorded. Sepsis was defined according to the American College of Chest Physicians/Society of Critical Care Medicine Consensus Conference [18]. Sepsis was subcategorized by site (e.g., pulmonary, bloodstream). Sepsis due to an OI was separately subcategorized.

Illness severity was captured by the APACHE II score within 24 hours of ICU admission. Organ failure/support was collected, including receipt of mechanical ventilation, number of days of ventilation, renal replacement therapy, and presence of shock (defined as the administration of vasopressors during the first 24 hours of ICU admission for persistent hypotension despite adequate fluid resuscitation). The first lactate dehydrogenase (LDH) and serum albumin measured in ICU were also recorded.

Our primary outcome was 30-day mortality (from time of ICU admission). Secondary outcomes included other patient-centered outcomes (ICU and in-hospital mortality) and health services utilization (receipt of organ support, ICU length of stay [LOS]).

In cases where patients were transferred between ICUs due to capacity limitations, ICU admissions were combined and considered a single admission. We only analyzed the first ICU admission for each patient; multiple admissions were excluded.

### 2.3. Statistical Analysis

Baseline characteristics of the cohort were expressed as means with standard deviations (SD) or medians with interquartile ranges (IQR) for continuous variables. Categorical variables were expressed as a number and percentage.

HIV-infected survivors and nonsurvivors were then compared using Chi-square or Fisher's exact tests for categorical variables and *t*-tests or Mann–Whitney *U* tests for continuous variables. Tests were two-tailed with an alpha of 0.05. Variables of clinical significance (age, sex, CD4 count, and PVL) and those with *p* values ≤ 0.1 in univariate analyses were included in multivariable Cox proportional hazards regression models to evaluate their association with mortality. For nonnormal continuous variables, we performed transformations to normalize the data. If no significant changes were noted, variables were left untransformed for ease of interpretation. Adjusted hazard ratios, 95% confidence intervals (95% CI), and *p* values for each variable were calculated. Proportional hazards model assumptions were checked using time-covariate interaction terms.

Our preplanned sensitivity analyses included entering the following variables to our primary model: albumin and LDH, hospital site, and admission date (to examine for changes over time the study period was split into two equal time periods: July 1, 2002–July 15, 2008 and July 16, 2008–July 31, 2014).

Statistical analyses were performed using IBM SPSS Statistics, Version 23.0 (Armonk, NY, IBM Corp.).

## 3. Results

### 3.1. Baseline Characteristics

**Figure 1:**
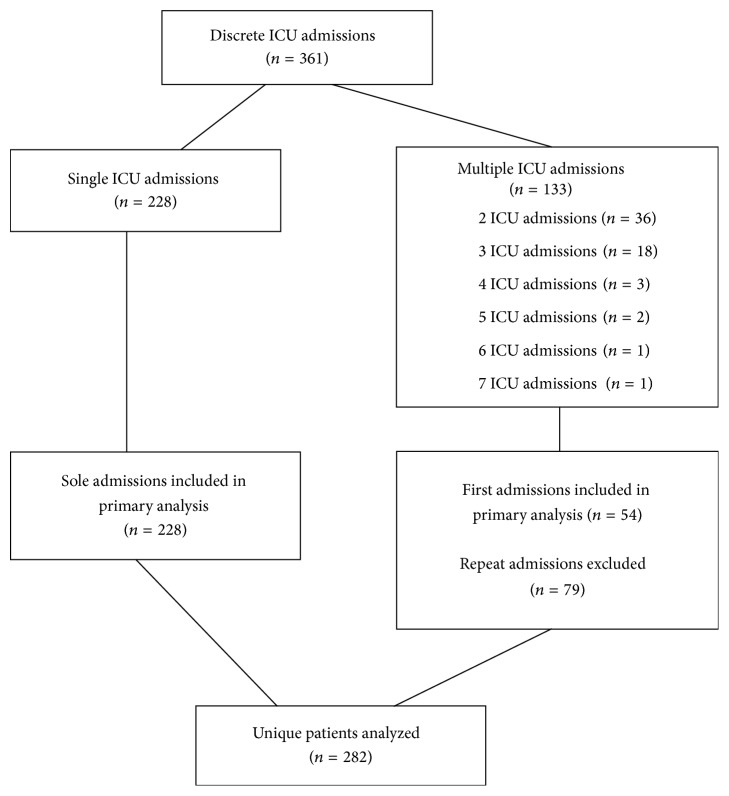
Patient flow sheet.

**Figure 2:**
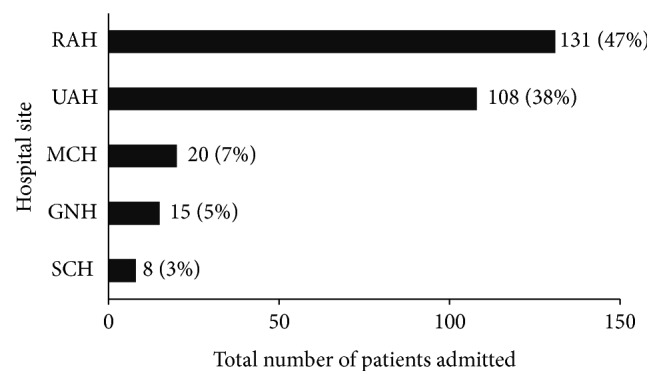
Number of patients by hospital site. SCH, Sturgeon Community Hospital; GNH, Grey Nun's Community Hospital; MCH, Misericordia Community Hospital; UAH, University of Alberta Hospital; RAH, Royal Alexandra Hospital.

Over the study period, the total number of ICU admissions in the region was 42632, of which 282 HIV-infected patients were admitted to ICU 366 times. Five admissions were inter-ICU transfers and were combined as one admission, leaving 361 discrete admissions. Fifty-four patients had multiple ICU admissions (Figure 1). Only the first admission for each of the 282 distinct patients was subsequently included in our analyses. The incidence of HIV-infected first admissions over the study period was 6.6 (95% CI 5.9–7.4) per 1000 admissions. The two academic/tertiary centers admitted the majority of patients (Figure 2).

Baseline characteristics of the cohort are shown in Table 1. Patients were young, with a mean patient age of 44 (SD 10) years. A large number of patients [134/282 (49%)] were aboriginal and HCV coinfected [153/282 (54%)]. Of the 181/282 (64%) who were HCV antibody positive, HCV RNA was negative for 28/181 (16%) and unavailable for 60/181 (33%). Thirteen percent (37/282) had preexisting cirrhosis. Alcohol and drug abuse history was very common in this population [184/282 (65%)].

### 3.2. HIV Control

**Figure 3:**
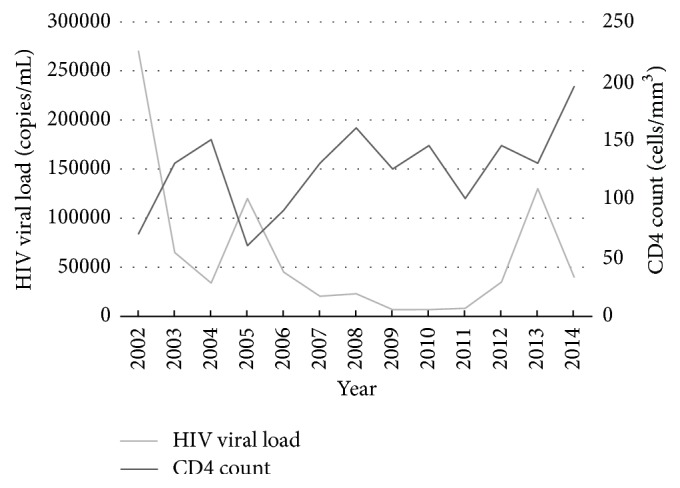
Trend in HIV viral load and CD4 count over time.

The median CD4 count was 125 cells/mm^3^ (IQR 30–300 cells/mm^3^), with a median CD4 percentage of 15 (IQR 6–25). The CD4 count was ≤200 cells/mm^3^ (AIDS-defining) in 167/282 (59%) patients. Median HIV PVL was 28,000 copies/mL (IQR 110–270,000 copies/mL). Over the study period, the median CD4 count trended up and the HIV PVL trended down (Figure 3).

HIV infection was newly diagnosed in 45/282 (16%) of patients. Patients newly diagnosed with HIV at the time of critical illness were older (mean age 49 [SD 11] versus 43 [SD 9] years; *p* = 0.001) and more likely to be male (78% versus 57%; *p* = 0.008) and had lower median CD4 counts (40 [IQR 10–78] versus 175 [IQR 53–338]; *p* < 0.001) and higher median PVL (395000 [IQR 110000–750000] versus 8200 [IQR 0–130000], *p* < 0.001) compared with those known to have HIV infection at ICU admission.

Of the 237 patients with known HIV infection at the time ICU admission, 98/237 patients (41%) were prescribed cART. The median CD4 count and percentage in patients on therapy were 230 cells/mm^3^ (IQR 90–420 cells/mm^3^) and 19% (IQR 10–29), respectively. PVL was suppressed in only 79/237 (33%) of patients overall, but 79/98 (81%) of patients prescribed cART had suppressed PVL. Patients with suppressed PVL had significantly higher median CD4 counts and percentages (260 cells/mm^3^ [IQR 130–465 cells/mm^3^; *p* < 0.001] and 22% [IQR 16–33]; *p* < 0.001) compared to nonsuppressed.

The mean duration of HIV infection for patients with a prior HIV diagnosis was 6.0 years (SD 5.5). At the time of ICU admission, 33/237 (14%) with known HIV infection did not have CD4 count and PVL testing performed within the preceding six months, suggesting a lack of engagement in care.

### 3.3. ICU Admission Diagnosis

The most common ICU admission diagnosis was sepsis (189/282; 64%). Sources of sepsis can be found in Table 2. Of note, pulmonary infections were the most common (127/189; 67%). These included pneumonia, empyema,* Pneumocystis jirovecii* pneumonia (PJP), and aspiration.

Of the 46 patients admitted to ICU with OI, 42/46 (91%) had PJP. Other OIs included cryptococcal meningitis, cerebral toxoplasmosis, and disseminated tuberculosis. Most (41/46; 89%) patients were not receiving cART and only one patient was taking PJP prophylaxis. Median absolute CD4 count among patients with OIs was 20 cells/mm^3^ [IQR 10–50]. Of these patients, 23/46 (50%) had newly diagnosed HIV.

### 3.4. Mortality Data

Crude 30-day mortality was 55/282 (20%) (Tables 1 and 3). Survivors were more likely to have newly diagnosed HIV (41/227; 18%) compared with nonsurvivors (4/55; 7%; *p* = 0.05). Additionally, survivors had a shorter average duration of HIV infection (5.5 years) compared with nonsurvivors (7.9 years). HIV disease control, including CD4 count and PVL, was similar between survivors and nonsurvivors. Mean APACHE II score was greater for nonsurvivors [28 (SD 9)] than for survivors [21 (SD 7)]. Among those with data, survivors had lower median LDH values compared with nonsurvivors [281 (IQR 168–456) versus 337 (234–603) U/L; *p* = 0.023] and higher mean albumin values [25 (SD 7) versus 22 (SD 6) g/L; *p* = 0.004].

### 3.5. Changes over Time

The incidence of HIV infection ranged from 4.2 to 10.0 per 1000 ICU admissions per year, with no trend over time (*p* = 0.78). During period 1, (July 1, 2002–July 15, 2008) HIV incidence was 7.0 (95% CI 5.8–8.2) per 1000 ICU admissions. Of these admissions, 84/136 (62%) were for sepsis. 30-day mortality in this early cohort was 31/136 (23%), ICU mortality was 25/136 (18%), and hospital mortality was 40/136 (29%).

During period 2 (July 16, 2008–July 31, 2014), the incidence of HIV was 6.4 (95% CI 5.4–7.5) per 1000 ICU admissions. A comparable number of patients were admitted with sepsis [96/146 (66%)] and outcomes were similar for 30-day mortality [25/146 (17%)], ICU mortality [22/146 (15%)], and hospital mortality [32/146 (29%)].

There were no differences in mortality by time period (early versus late) when the study period was split into two equal time periods as described above (aHR 0.71; 95% CI 0.37–1.36; *p* = 0.3 for the more contemporary period compared to the earlier time period) in multivariable modeling.

### 3.6. Survival and Sensitivity Analyses

Cox regression modeling results are shown in Table 4. Preexisting cirrhosis (aHR 2.3; 95% CI 1.1–4.7; *p* = 0.024), CAD (aHR 7.0; 95% CI 2–22; *p* = 0.001), duration of HIV infection (aHR 1.07 per year of infection; 95% CI 1.02–1.13; *p* = 0.01), and APACHE II score (aHR 1.12; 95% CI 1.08–1.16; *p* < 0.001) were independently associated with 30-day mortality. Of note, CD4 count and PVL were not associated with 30-day mortality.

In our first sensitivity analysis, we explored the association between albumin and 30-day mortality by adding albumin to our primary model. 30/282 (11%) of patients with missing data were excluded from the analysis. Albumin was not independently associated with mortality (aHR 0.95; 95% CI 0.90–1.0; *p* = 0.059).

In our second sensitivity analysis, we examined LDH as a predictor of mortality. There were a large number (92/282; 33%) of patients with missing values. Higher LDH, however, was associated with higher 30-day mortality (aHR 1.003 per 10-unit increase; 95% CI 1.000–1.006; *p* = 0.047).

Third, there were no differences in 30-day mortality based on hospital site. Proportional hazards assumptions were met for all models.

## 4. Discussion

In this study, we describe the epidemiology of and outcomes in a population-based cohort of critically ill HIV-infected patients in the post-cART era. Similar to previously published data, our patient population was young (mean age 44 years) [5, 12, 19]. Nearly half of patients were aboriginal and the prevalence of substance use and HCV coinfection were high. Aboriginal patients are disproportionately represented in the HIV-infected population in northern Alberta, with a prevalence of approximately 27% (Houston SC, personal communication, 2016 Apr 20). The high incidence of HIV infection in aboriginal peoples is unfortunately not restricted to our geographic region [20–22]. Furthermore, aboriginal patients are admitted to ICU at a rate that exceeds their local population prevalence [23]. Interestingly, however, age, sex, ethnicity, substance use, and viral coinfection were not associated with mortality. We postulate that the high prevalence of some of these factors, such as substance abuse, may reflect a higher relative risk of ICU admission compared to other HIV-infected patients [24, 25].

HIV guidelines [26] currently recommend cART initiation in all patients with HIV infection, regardless of CD4 count. Despite these widespread recommendations, only 41% of known HIV-infected patients in this study were on cART at the time of admission. We believe this reflects social instability and disengagement with care in our population and an opportunity for intervention. The low proportion of patients receiving cART at the time of ICU admission is consistent with previous studies [5, 12]. We recognize that there is likely a selection bias, in that critically ill HIV-infected patients may reflect a subset of the broader HIV-infected population that is less engaged in care and less likely to be on cART. Despite this and similar to prior studies, CD4 count and PVL were not independently associated with short-term mortality [3, 9, 27–30]. Instead, acute illness severity was strongly associated with mortality in our study, an effect which has been demonstrated in several other studies [6, 9, 31–33].

Consistent with the lack of association between CD4 count or PVL and mortality, receipt of cART at the time of ICU admission was not associated with short-term mortality. Several retrospective studies have had similar findings [5, 13, 27, 30, 34, 35]. Only two recent studies demonstrated a survival benefit with cART [6, 9]. While HIV control may predict survival over the longer term [31], early death appears to be largely driven by acute illness severity and not markers of HIV control.

Interestingly, longer duration of HIV infection was independently associated with 30-day mortality even after adjustment for CD4 count and PVL. We postulate that patients with longer duration of HIV infection may have had longer antiretroviral exposure and subsequent toxicities (e.g., CAD with protease inhibitor use [36]). Furthermore, the prolonged cumulative inflammatory effects of HIV infection [14, 37, 38] may translate to increased susceptibility to and mortality following an episode of critical illness.

Opportunistic infection was relatively uncommon in our cohort (17%), consistent with recent data demonstrating that HIV-related admissions have declined in the cART era [39, 40]. The most common OI was PJP (87%). Not surprisingly, the median CD4 count in patients with OIs was extremely low (20 cells/mm^3^). Importantly, half of patients with OIs had newly diagnosed HIV infection.

Neither sepsis nor shock was significantly associated with 30-day mortality. This is in contrast to many previous studies, which found that sepsis was a significant predictor of mortality in critically ill patients with HIV infection [5, 12, 19, 31, 41–43]. The high incidence of sepsis in our population may explain our inability to detect a mortality difference, if one truly exists.

Preexisting CAD and cirrhosis were both independently associated with mortality. The association between cirrhosis and mortality in HIV-infected patients has been previously recognized [44]. Previous studies have also shown that HIV/HCV coinfected patients have a higher risk of ICU mortality compared with HIV monoinfected patients [19]; however we did not observe this. While CAD has been recognized to increase the likelihood of ICU admission in hospitalized patients [43], no previous correlation between preexisting CAD and ICU mortality has been demonstrated in HIV-infected populations. However, given the low number of patients with CAD in our cohort, this requires further validation.

Despite its strengths, our study has several limitations. First, it is observational and retrospective; however follow-up was complete and mortality data were available for all patients. In addition, we risk-adjusted to minimize confounding. Second, although our study was multicentered, it was restricted to a single geographic region thereby limiting the generalizability of our findings. Third, some baseline variable data were missing. Although minimal data were missing for some variables (e.g., 2/282 [<1%] for CD4 count), this was more substantial for other variables (e.g., 84/282 [30%] for LDH), which limited our ability to include all variables in our analyses. Fourth, the APACHE II score was developed in 1985 and is considered by some to be somewhat outdated. However, it remains one of the best-known and widely used scores in North America to predict mortality of critically ill patients. The APACHE II score has been validated in a number of ICU populations, including Canadian patients [45, 46]. Furthermore, the APACHE II score has been found to be superior to other scores such as the Simplified Acute Physiology Score (SAPS II) and the Sepsis-related Organ Failure Assessment (SOFA) score [47] in predicting ICU mortality. Although more contemporary APACHE scores are available, these newer scores are more onerous in terms of data collection and depend upon proprietary software. For these reasons, many ICUs continue to calculate the APACHE II score at admission and therefore these data are more commonly available for retrospective use. Fifth, some data (such as CD4 count) may have been influenced by acute illness and therefore not reflective of baseline premorbid state or HIV control. Sixth, adherence to cART and/or prophylaxis could not be confirmed. Therefore, we could not assume that an unsuppressed HIV PVL was due to nonadherence versus other factors (such as drug resistance and/or recent cART initiation). That said, prior studies have found higher CD4 counts and lower HIV PVLs among patients receiving ARVs, even when treatment adherence has not been defined or collected [15]. Seventh, defining suppressed HIV PVL as <200 copies/mL to account for changing assay sensitivity over time introduced some heterogeneity into the subset of patients identified as having suppressed PVL. Patients with very low level viral replication, associated with increased risk of treatment failure and higher levels of inflammation, were grouped with the patients with undetectable PVL. Eighth, limiting our primary analysis to the first ICU admission for each patient excluded all data from subsequent admissions; however, we chose this approach to avoid intrasubject correlation. Lastly, we recognize that selection bias in our publically funded health care system is unavoidable. It remains at the discretion of the intensivist to decline ICU admission to patients when they perceive critical care to be futile. Therefore, patients at high risk of mortality or perceived poor prognosis may not be offered ICU admission. In particular, patients who have been not able to engage in care or adhere to cART prior to critical illness may be perceived as having poor long-term prognosis. Our study did not capture HIV-infected patients who were critically ill but were not admitted to ICU.

We believe our data have implications for health care providers. Given that short-term outcomes appear to be predominantly driven by severity of acute illness, not HIV control, we would advocate that HIV patients receive similar and appropriately aggressive treatments as other critically ill patients. In addition, for those previously diagnosed with HIV infection but not engaged in care, an admission to ICU represents an opportunity for reengagement.

## 5. Conclusions

In this Canadian population-based cohort of HIV-infected critically ill patients, severity of critical illness and non-HIV-related comorbidities were independently associated with 30-day mortality, while specific measures of HIV control were not. Longer duration of HIV infection was associated with increased mortality, suggesting there may be subtle effects of prolonged HIV infection and/or prolonged cART exposure that may modify survival. The association between newly diagnosed HIV and higher survival reinforces the importance of critical care support, particularly in patients yet to have the opportunity to engage in care. However, even in patients with advanced HIV infection, the fact that short-term outcomes appear to be predominantly driven by severity of acute illness rather than by HIV control lends support to the current practice of delivering similarly aggressive care to HIV-infected patients as to other critically ill patients. The low proportion of critically ill patients with known HIV on cART suggests an opportunity for reengagement during recovery from acute illness.

## Figures and Tables

**Table 1:** Baseline characteristics and univariate analyses.

Variable	All patients	Death at 30 days		*p* value
		No	Yes	
	*n* = 282	*n* = 227	*n* = 55	
	(100%)	(80.5%)	(19.5%)	(Chi-square)
Age in years (mean, SD)	44.2 (9.9)	43.8 (10)	45.8 (9.3)	0.19
Male gender	169 (59.9)	134 (59.0)	35 (63.6)	0.11
*Ethnicity*				0.11
Aboriginal	134 (47.5)	111 (48.9)	23 (41.8)	0.35
Caucasian	115 (40.8)	90 (39.6)	25 (45.5)	0.43
Black	17 (6.0)	15 (6.6)	2 (3.6)	0.41
Hospital site				0.87
*Comorbidities*				
HCV coinfection^∧^	153 (54.4)	131 (53.5)	32 (58.2)	0.54
HBV coinfection^∧^	17 (6.0)	14 (6.2)	3 (5.5)	1.00
HBV/HCV^∧^	6 (2.1)			
Chronic lung disease	57 (20.2)	46 (20.3)	11 (20.0)	0.97
Malignancy	21 (7.4)	15 (6.6)	6 (10.9)	0.26
Psychiatric disease	68 (24.1)	57 (25.1)	11 (20.0)	0.43
Cirrhosis	37 (13.1)	25 (11.0)	12 (21.8)	**0.033**
CAD	10 (3.5)	5 (2.2)	5 (9.1)	**0.027**
Addiction	184 (65.2)	148 (65.2)	36 (65.5)	0.97
*HIV risk factor*				
PWID	169 (59.9)	137 (60.4)	32 (58.2)	0.77
MSM	27 (9.6)	18 (7.9)	9 (16.4)	0.056
Heterosexual	186 (66.0)	150 (66.1)	36 (65.5)	0.93
New diagnosis of HIV	45 (16.0)	41 (18.1)	4 (7.3)	**0.050**
Duration of HIV (years, mean, SD)^∧^	6.0 (5.5)	5.5 (5.5)	7.9 (5.2)	**0.003**
cART at ICU admission	98 (34.8)	75 (33.0)	23 (41.8)	0.22
PI-based regimen	53 (18.8)	41 (18.1)	12 (21.8)	0.52
NNRTI-based regimen	50 (17.7)	39 (17.2)	11 (20.0)	0.62
PJP prophylaxis at ICU admission	40 (14.2)	32 (14.1)	8 (14.5)	0.93
CD4 count, cells/mm^3^ (median, IQR)^∧^	125 (30–300)	120 (35–306)	140 (20–290)	0.53
CD4 percentage (median, IQR)^∧^	15 (6–25)	14 (6–24.75)	15 (3–26)	0.48
HIV viral load, copies/mL (median, IQR)^∧^	28000 (110–270000)	28000 (130–291864)	28000 (0–197500)	0.40
HIV viral load suppressed^∧^	78 (28.4)	60 (27.1)	18 (33.3)	0.37
History of OI	90 (31.9)	68 (30.0)	22 (40.0)	0.15
*Admission diagnosis*				
Respiratory failure	10 (3.5)	9 (4)	1 (1.8)	0.69
Sepsis	189 (63.8)	139 (61.2)	41 (74.5)	0.065
Trauma	23 (8.2)	22 (9.7)	1 (1.8)	0.057
Overdose	32 (11.3)	29 (12.8)	3 (5.5)	0.13
APACHE II score (mean, SD)	22.1 (7.8)	20.7 (6.9)	27.7 (8.6)	**<0.001**
LDH (median, IQR)^∧^	292 (187–471)	281 (168–456)	337 (234–603)	**0.023**
Albumin (mean, SD)^∧^	24.3 (7.2)	25 (7.4)	21.8 (5.9)	**0.004**
IMV	213 (75.5)	167 (73.6)	46 (83.6)	0.12
Mechanical ventilation days (median, IQR)	3 (1–7)	3 (1–7)	3 (1–6)	0.76
Shock	133 (47.2)	98 (43.2)	35 (63.6)	**0.006**
AKI requiring RRT	35 (12.4)	25 (11)	10 (18.2)	0.15

HCV, hepatitis C virus; HBV, hepatitis B virus; CAD, coronary artery disease; PWID, people who inject drugs; MSM, men who have sex with men; ARV, antiretroviral; PI, protease inhibitor; NNRTI, nonnucleoside reverse transcriptase inhibitor; PJP, *Pneumocystis jirovecii*; PVL, plasma viral load; LDH, lactate dehydrogenase; IMV, invasive mechanical ventilation; AKI, acute kidney injury; RRT, renal replacement therapy; ICU, intensive care unit; LOS, length of stay.

^∧^Missing data; bold, statistically significant.

**Table 2:** Sources of sepsis.

Source	All patients with sepsis *N* = 189 (100%)
Pulmonary	127 (67)
Bloodstream (including infective endocarditis)	18 (10)
Soft tissue or musculoskeletal (e.g., cellulitis and necrotizing fasciitis)	10 (5)
Gastrointestinal (e.g., perforated viscus and *Clostridium difficile* infection)	8 (4)
Urinary tract	6 (3)
Central nervous system	4 (2)
Miscellaneous	12 (6)
Unknown	4 (2)

**Table 3:** ICU outcomes and univariate analyses.

Variable	All patients	Death at 30 days		*p* value
		No	Yes	
	*n* = 282	*n* = 227	*n* = 55	
	(100%)	(80.5%)	(19.5%)	(Chi-square)
ICU LOS in hours (median, IQR)	113 (53–237)	113 (57–228)	97 (27–289)	0.21
Death in ICU	46 (16.3)			
Death in hospital	70 (24.8)			
Death at 30 days	55 (19.5)			

**Table 4:** Survival analysis for predictors of 30-day mortality.

Variable	aHR (95% CI)	*p* value
Age	1.00 (0.96–1.03)	0.75
Male gender	1.16 (0.62–2.16)	0.64
*Cirrhosis*	2.30 (1.12–4.73)	*0.024*
*CAD*	6.98 (2.20–22.13)	*0.001*
Men who have sex with men	1.30 (0.54–3.11)	0.55
New diagnosis of HIV	0.67 (0.20–2.26)	0.52
*Duration of HIV (years)*	1.07 (1.02–1.13)	*0.010*
CD4 count (per 10 cells/mm^3^)	1.00 (0.99–1.01)	0.98
HIV viral load (per 1000 copies/mL)	1.00 (1.00–1.00)	0.68
*APACHE II*	1.12 (1.08–1.16)	<*0.001*
Admission diagnosis		0.46
Shock	0.80 (0.40–1.61)	0.54
